# Genotype-phenotype correlation of TGFBI corneal dystrophies in Polish patients

**Published:** 2011-08-30

**Authors:** Anna K. Nowińska, Edward Wylegala, Dominika A. Janiszewska, Dariusz Dobrowolski, Pasquale Aragona, Anna M. Roszkowska, Domenico Puzzolo

**Affiliations:** 1 Department of Ophthalmology, District Railway Hospital, Katowice, Poland; 2 Department of Biophysics, University of Silesia, Katowice, Poland; 3 Department of Ophthalmology Saint Barbara Hospital, Sosnowiec, Poland; 4 Department of Surgical Specialties, Section of Ophthalmology, University of Messina, Messina, Italy; 5 Department of Biomorphology and Biotechnologies, University of Messina, Messina, Italy

## Abstract

**Purpose:**

To analyze genotype-phenotype correlation in patients originating from Polish population with the transforming growth factor beta induced (TGFBI) corneal dystrophies.

**Methods:**

Sixty affected and 31 unaffected individuals from 15 unrelated Polish families were included in the study. The clinical diagnosis was based on the slit-lamp exam, 1310 nm time domain and 1310 nm swept source spectral domain optical coherence tomography (OCT). Histopathologic analysis was performed on 10 available corneal buttons. Exons of the *TGFBI* gene were screened for mutations with polymerase chain reaction (PCR) and direct DNA sequencing.

**Results:**

We found the lattice phenotype dominant compared to the granular one in the Polish population (41:16 patients; lattice:granular). We identified five distinct mutations responsible for TGFBI corneal dystrophies (R124R, R124H, R555W, R555Q, and H626R). There was a strong genotype-phenotype correlation in the case of R124R and R555W mutations, while there was a distinct phenotypic heterogeneity in the case of the H626R mutation. OCT analysis revealed that the reflectivity, location and pattern of the corneal deposits were different among the TGFBI corneal dystrophies. The advantage of spectral swept source OCT over time-domain OCT scans is a more distinct visualization of the Bowman’s layer area and deposits located under the epithelium.

**Conclusions:**

This study underlines the role of comprehensive phenotype-genotype analysis in TGFBI corneal dystrophies, describes for the first time the TGFBI mutation spectrum in a Polish population and reveals phenotypic heterogeneity in the case of the H626R mutation.

## Introduction

Corneal dystrophies as a group of inherited, bilateral, symmetric, slowly progressive corneal diseases without any relationship to environmental or systemic factors have been mapped to 10 different chromosomes: 1, 2, 5, 9, 10, 12, 16, 17, 20, and X [1]. Knowledge regarding the molecular basis of corneal dystrophies is still evolving, as underlined in the IC3D classification (the International Committee for Classification of Corneal Dystrophies).

Transforming growth factor-β induced gene corneal dystrophies (TGFBI; OMIM 601692) are a heterogeneous group of dystrophies, including epithelial basement membrane corneal dystrophy (EBMD; OMIM 121820), Bowman’s layer dystrophies: Thiel-Behnke corneal dystrophy (TBCD; OMIM 602082), Reis-Bűcklers – granular corneal dystrophy type III (GCD3; OMIM 608470), and the stromal dystrophies: lattice corneal dystrophy type I (LCDI; OMIM 122200) and variants (LCDvariants), granular corneal dystrophy type I (GCDI, OMIM 121900) and II (granular-lattice, Avellino – GCD2; OMIM 607541).

More than 50 distinct disease-causing mutations have been identified in the *TGFBI* gene [2]. Different population-wide studies revealed two mutational hot spots at codons R124 and R555 [3-5]. Researchers have established that TGFBI corneal dystrophies are inherited with complete penetrance and dominance and certain *TGFBI* mutations are correlated with a specific phenotype. Researchers have also established that the R124C mutation is associated with lattice corneal dystrophy type I, the R124L mutation is associated with Reis-Bucklers corneal dystrophy, the R555W mutation is associated with granular corneal dystrophy type I, the R555Q mutation is associated with Thiel-Behnke corneal dystrophy, whereas granular corneal dystrophy type II is associated with the R124H mutation. Later studies reported cases of increased severity of deposits, multiple recurrences and earlier onset for patients bearing homozygous mutations suggesting a semi-dominant inheritance pattern [6-11]. Cases of reduced penetrance have also been reported [12,13]. Several reports underlined the phenotypic and mutational heterogeneity according to rare mutations and, in recent years, to common *TGFBI* mutations [14-20].

Although molecular studies in differential diagnosis of TGFBI dystrophies have been used increasingly in clinical practice during recent years, phenotype analysis and histopathologic examination remain the “gold standard” for determining the type of corneal dystrophy. Additionally, in recent years, corneal imaging techniques have been developed that allow in vivo corneal morphology and morphometry assessment to analyze the disease progression and determine the method of treatment. Confocal microscopy, invented in 1955 and developed in the nineties, has been used for high-resolution imaging and provides en face images of the corneal layers [21]. The IC3D classification system contains a description of confocal microscopy images of all corneal dystrophies. Optical coherence tomography (OCT) is a high-speed, high-resolution, non-contact optical imaging technique introduced in 1991 for cross-sectional imaging in biologic systems [22]. Anterior eye segment imaging with the 830 nm light wavelength OCT was demonstrated in 1994 [23]. Introduction of transscleral anterior eye segment imaging was achieved by changing the light wavelength from 830 nm to 1310 nm [24]. In recent years, OCT technology has been revolutionized by the development of spectral domain technology, which provides an increased signal-to-noise ratio and increased robustness compared with time domain OCT [25]. OCT is proven to provide reliable anterior eye segment parameters measurements characterized by good repeatability and reproducibility [26].

We report, for the first time, the TGFBI mutation spectrum and genotype-phenotype correlation integrating the slit-lamp exam, corneal morphology assessment with two OCT systems, histological exam and genetic analysis of patients encompassing a population in Poland.

## Methods

The study was approved by the Ethics Committee of the Medical University of Silesia, Katowice, Poland (SUM NN-6501–175/I/07). All patients had to sign informed consent forms before undergoing any study procedure. A family was included in the study only if at least two family members had been diagnosed with TGFBI corneal dystrophy.

### Patients

Sixty affected and 31 unaffected individuals (50 women and 41 men) from 15 unrelated Polish families were included in the study (family F1-F15). The mean age of the patients was 46 years (±19 years). The mean number of examined patients was 5 per family (ranging from 3 to 11 patients). Seventeen patients had undergone a keratoplasty procedure in the past, while 9 patients underwent keratoplasty while this study was performed.

### Phenotype analysis

A detailed family history was collected, and pedigree charts were constructed for all patients. We analyzed the age at onset of symptoms and type of symptoms reported by patients. The clinical examination consisted of visual acuity, slit-lamp biomicroscopy with photography, time domain and spectral domain optical coherence tomography.

We analyzed corneal change patterns, location, symmetry and progression with age. In addition, heterogeneity of corneal morphology among patients with the same TGFBI mutation was assessed.

### Optical coherence tomography

Anterior segment imaging was performed by one observer. We used two anterior segment optical coherence systems: time domain OCT (Visante OCT; Carl Zeiss Meditec, Inc., Dublin, CA) and 1310 nm swept source spectral domain OCT (SS-1000 CASIA; Tomey Co., Nagoya, Japan). During the time domain OCT exam, corneal morphology was assessed on high-resolution corneal quad scans (10×3 mm; 4×512 A-scans). During the swept source spectral OCT exam, corneal morphology was assessed with radial scans (10×4 mm; 128×512 A-scans).

### Histologic examination

The 10 available corneal buttons from 9 patients (6 without the Descemet membrane and posterior corneal stroma after deep anterior lamellar keratoplasty and 4 whole corneal buttons after penetrating keratoplasty) were processed by standard methods involving sectioning of the tissue samples. Corneal sections were stained with hematoxylin and eosin (HE), periodic acid-Schiff (PAS), Alcian blue, Masson's trichrome, and Congo red and further analyzed with light microscopy for the presence of amyloid and/or hyaline deposits. The presence of amyloids was confirmed by birefringence when viewed under polarized light.

### DNA collection, isolation, amplification and sequencing

DNA was isolated from dry blood samples collected on FTA^®^ cards (Whatmann, Maidstone, UK). For DNA isolation, a 2.0-mm-diameter disc was punched and collected into a sterile Eppendorf tube. DNA was isolated by using the lysis and neutralization solutions from REDExtract-N-Amp™ Blood PCR Kit (Sigma-Aldrich, St. Louis, MO) according to the manufacturer’s protocol. Exons were amplified by polymerase chain reaction (PCR) with the primers published by Munier et al. [3] (Table 1). The PCR product was purified and directly submitted for DNA sequencing. In case of 15 probands, we started the analysis with screening exon 4 and exon 12 of the *TGFBI* gene, where hot spot sites for mutations have been previously reported [2-5]. If no mutation was revealed, we screened *TGFBI* exon by exon until the mutation was defined. After that, in case of other family members, we analyzed the exons for which the mutation was defined for the proband.

**Table 1:** Table showing *TGFBI* primer sequences.

**Exon**	**Sequence of forward and reverse primer**	**Annealing temp (°C)**	**Product size (bp)**
1	5’-CCGCTCGCAGCTTACTTAAC-3’	58	362
	5’-AGCGCTCCATGCTGCAAGGT-3’		
2	5’-GTGGACGTGCTGATCATCTT-3’	55	170
	5’-TCCTGGCTGGTTACAGATAC-3’		
3	5’-GCTGTGGAGGCAACTTAGTG-3’	55	280
	5’-GAGAATGCCATGTCCTTGTG-3’		
4	5’-CCCCAGAGGCCATCCCTCCT-3’	58	226
	5’-CCGGGCAGACGGAGGTCATC-3’		
5	5’-TAAACACAGAGTCTGCAGCC-3’	55	260
	5’-TTCATTATGCACCAAGGGCC-3’		
6	5’-TGTGTTGACTGCTCATCCTT-3’	55	316
	5’-CATTCAGGGGAACCTGCTCT-3’		
7	5’-AAGTGTGCCAAGTTGACCTC-3’	55	588
	5’-GGCAGGTGGTATGTTCATCT-3’		
8	5’-AGAAGGCGAGGAGGATCTGG-3’	55	508
	5’-CAGTGGCCGAGAAGCTGTGA-3’		
9	5’-CATTCCTGCTGATGTGTGTCATGC-3’	55	315
	5’-GGGTGCTGTAAATCGGAGAGTGTTG-3’		
10	5’-TCTGGACCTAACCATCACCC-3’	55	206
	5’-CAGGAGCATGATTAGGACC-3’		
11	5’-CTCGTGGGAGTATAACCAGT-3’	55	220
	5’-TGGGCAGAAGCTCCACCCGG-3’		
12	5’-CATTCCAGTGGCCTGGACTCTACTATC-3’	58	340
	5’-GGGGCCCTGAGGGATCACTACTT-3’		
13	5’-CCTCCTTGACCAGGCTAATTAC-3’	55	300
	5’-GGCTGCAACTTGAAGGTTGTG-3’		
14	5’-CTGTTCAGTAAACACTTGCT-3’	55	265
	5’-CTCTCCACCAACTGCCACAT-3’		
15	5’-ACAGCATCTCACCTCAGTGT-3’	55	360
	5’-AACCTAGCAGGCATCTTACC-3’		
16	5’-GCTTGCACAACTTATGTCTG-3’	55	251
	5’-CAGGTCTGCAATGACTTC-3’		
17	5’-CCTGGTCCTTGAGATTCTGA-3’	55	489
	5’-GAGGCTGGATTGCTTGATTC-3’		

Based on data previously published by Munier et al. [3].

Direct sequencing was performed using the BigDye Terminator v3.1 (Applied Biosystems; Life Technologies, Foster City, CA). A 3730xl DNA Analyzer (Applied Biosystems) was used to collect the sequence data. Nucleotide sequences were compared with the published sequence of *TGFBI* (GenBank NM_000358) to analyze the sequence changes.

## Results

### Clinical findings

Clinical examination revealed 8 families (33 patients with clinical manifestation) with lattice corneal dystrophy type I (F1, F3, F5, F6, F12, F13, F14, and F15), 2 families (8 patients) with variants of lattice corneal dystrophy (F2 and F7), 3 families (14 patients) with granular corneal dystrophy type I (F4, F10, and F11), 1 family (2 patients) with granular corneal dystrophy type II (F9) and 1 family (3 patients) with Thiel-Behnke corneal dystrophy (F8).

The disease showed an autosomal dominant inheritance pattern in all families as revealed by pedigree analysis. The age of onset varied between different corneal dystrophies. In the case of patients with TBCD, LCDI, and GCDI and II, the onset of the disease was in the first and second decades of life. In patients with LCD variants, the onset was delayed to the fourth and fifth decades of life. Patients from all studied families reported recurrent erosion and visual impairment during the course of the disease.

### Molecular analysis

Sequencing of the *TGFBI* gene revealed a nucleotide transition in all affected and none of unaffected family members: c.1711G>A of exon 12 causing the R555Q mutation in Thiel-Behnke corneal dystrophy (F8), c.1710C>T of exon 12 causing the R555W mutation in granular corneal dystrophy type 1 (F4, F10, and F11); c.418G>A of exon 4 causing the R124H mutation in granular corneal dystrophy type 2 (F9), c.417C>T of exon 4 causing the R124C mutation in lattice corneal dystrophy type 1 (F1, F3, F5, F6, F12, F13, F14, and F15), and c.1924A>G of exon 14 causing the H626R mutation in variant of lattice corneal dystrophy (F2 and F7). Results of genetic analysis with comparison to corneal phenotypes are summarized in Table 2.

**Table 2:** Summary of genetic, slit lamp microscopy and optical coherence tomography exam results.

Corneal dystrophy	Family No. (F1-F15)	Number of patients	Exon	Nucleotide	TGFBI mutation detected	Slit lamp exam – corneal morphology	Optical coherence tomography - corneal morphology	Histology
GCDI	F4, F10, F11	14	12	1710C>T	R555W	Granular crumb-like anterior stromal deposits in the central cornea, with age becoming located deeper in the stroma	Focal granular hyperreflective changes in the area of Bowman layer and anterior to mid corneal stroma; with age, during fourth and fifth decade extending to Descemet’s membrane	MT+ve (3 corneal buttons from families F4, F10)
GCDII	F9	2	4	418G>A	R124H	Star- and disc- shaped stromal opacities in the central cornea	Highly reflective corneal opacities in anterior stroma accompanied with focal, deposits located in the mid stroma	CR+ve, MT+ve (1 corneal buton from family F9)
TBCD	F8	3	12	1711G>A	R555Q	Reticular pattern of corneal deposits in the anterior corneal part	Increased reflectivity and irregularity in the area of Bowman layer and anterior corneal stroma	-
LCDI	F1, F3, F5, F6, F12, F13, F14, F15	33	4	417C>T	R124C	Dots and lattice lines in the anterior to mid stroma in the central cornea	Diffuse areas of increased reflectivity in the area of Bowman layer and anterior to mid stroma	CR+ve (5 corneal buttons from families F1, F12,F15)
LCD variant	F2	4	14	1924A>G	H626R	Fragile, thin lines located in the central cornea	Corneal changes of increased reflectivity located at different depth, from the area of Bowman layer to Descemet’s membrane	CR +ve (1 corneal button from family F2)
LCD variant	F7	4	14	1924A>G	H626R	Thick, distinct lines extended from limbus to limbus, accompanied by stromal haze	Hyperreflective corneal changes located from anterior to posterior stroma. Some of corneal deposits located deep in the posterior stroma	-

GCDI - granular corneal dystrophy type I; GCDII - granular corneal dystrophy type II (granular-lattice); TBCD - Thiel-Behnke corneal dystrophy; LCDI – lattice corneal dystrophy type I; LCD variant – variant of lattice corneal dystrophy.

### Optical coherence tomography

**Figure 1:**
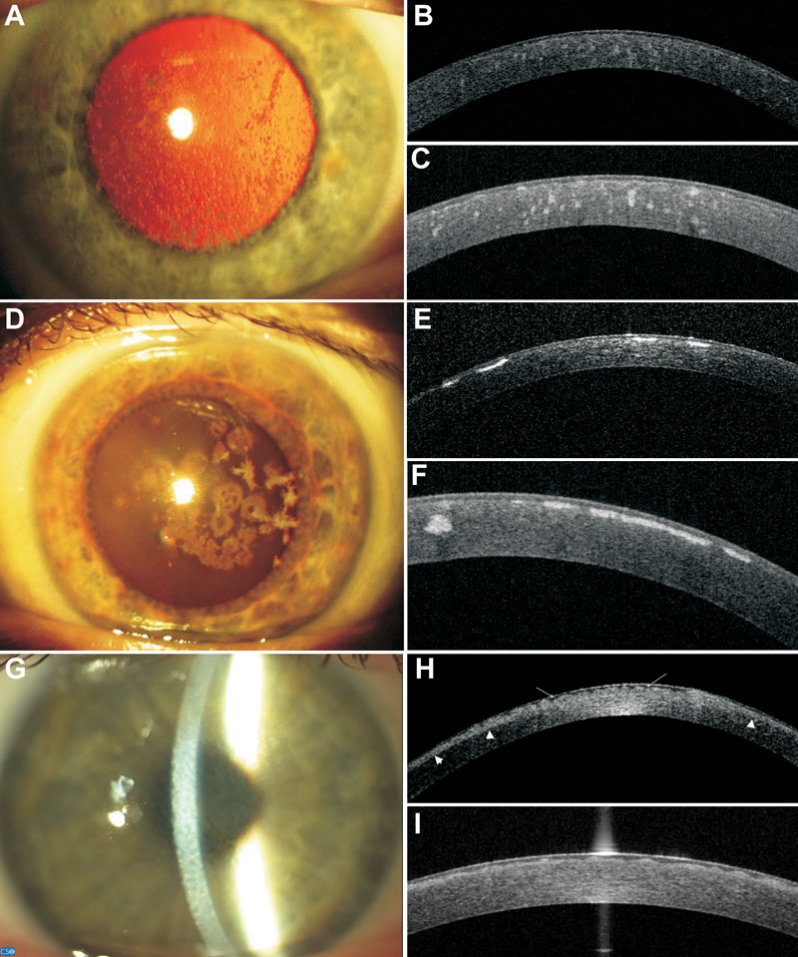
Representative images of slit-lamp photographs, 1310 nm time-domain and 1310 nm swept source spectral domain optical coherence tomography scans of patients with granular corneal dystrophy type I (family F4), granular corneal dystrophy type II (family F9), and Thiel-Behnke corneal dystrophy (family F8). **A**: Female patient (F4; 53 years). Slit-lamp photograph showing gray-white granular deposits located centrally, with clear intervening stroma. GCDI/ R555W mutation. **B**: Female patient (F4; 53 years). High-resolution corneal scan – 1310 nm time. domain OCT. Focal granular hyperreflective changes located at different depths within the corneal stroma. GCDI/ R555W mutation. **C**: Female patient (F4; 53 years). Radial scan-swept source 1310 nm spectral OCT. Focal granular hyperreflective changes located at different depths within the corneal stroma. The Bowman’s layer area shows a distinct irregularity. GCDI/ R555W mutation. **D**: Female patient (F9; 44 years). Slit-lamp photograph. Centrally located, multiform: star- and disc-shaped opacities. No lattice lines are visible, either on direct light nor on retroillumination. GCDII/ R124H mutation. **E**: Female patient (F9; 44 years). High-resolution corneal scan – 1310 nm time domain OCT. Highly reflective opacities with distinct borders located in the anterior corneal part. GCDII/ R124H mutation. **F**: Female patient (F9; 44 years). Radial scan-swept source 1310 nm spectral OCT. Highly reflective disc-shaped changes located in the anterior stroma, under the epithelium, involving Bowman’s layer. One hyperreflective granular opacity located deeper in the mid stroma. GCDII/ R124H mutation. **G**: Female patient (F8; 38 years). Slit-lamp photograph. Diffuse corneal changes showing reticular, “honeycomb” pattern located in the anterior corneal part. TBCD/ R555Q mutation. **H**: Female patient (F8; 38 years). High-resolution corneal scan – 1310 nm time domain OCT. The diffuse boarder of increased reflectivity in the anterior part of the cornea (arrowheads). In the Bowman’s layer area, there is a distinct irregularity due to corneal opacities (arrows). TBCD/ R555Q mutation. **I**: Female patient (F8; 38 years). Radial scan-swept source 1310 nm spectral OCT. Bowman’s layer is replaced by an irregular pattern of opacities. TBCD/R555Q mutation.

**Figure 2:**
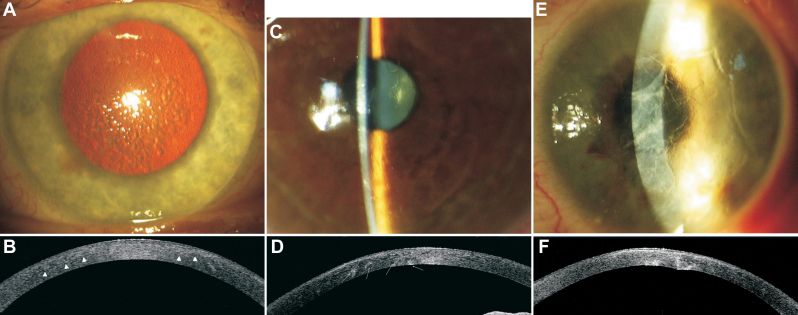
Representative images of slit-lamp photographs and 1310 nm time-domain optical coherence tomography scans of patients with lattice corneal dystrophy type I (family F1); lattice corneal dystrophy variants (families F2 and F7). There is a noticeable phenotypic heterogeneity between corneal morphology of lattice corneal dystrophy variants caring the same H626R mutation. **A**: Male patient (F1; 37 years). Slit-lamp retroillumination photograph showing diffuse multiple lattice lines. LCDI/R124C mutation. **B**: Male patient (F1; 37 years). High-resolution corneal scan – 1310 nm time. domain OCT. There is a diffuse border between the anterior part of increased reflectivity and normal corneal stroma (arrowheads). The areas of increased stromal reflectivity correspond with corneal opacities. LCDI/R124C mutation. **C**: Female patient (F2; 45 years). Slit-lamp photograph. Delicate, fragile, rare lattice lines located centrally. LCD variant/ H626 mutation. **D**: Female patient (F2; 45 years). High-resolution corneal scan – 1310 nm time. domain OCT. Opacities with increased reflectivity visible through the whole depth of the cornea. Some of the opacities are located in the posterior corneal part (arrows). LCD variant/H626 mutation. **E**: Female patient (F7; 48 years). Slit-lamp photograph. Thick, distinct lines accompanied by stromal haze extended from limbus to limbus. LCD variant/H626 mutation. Note the distinct heterogeneity compared to Figure 2C. **F**: Female patient (F7; 48 years). High-resolution corneal scan – 1310 nm time. domain OCT. Opacities with increased reflectivity located mainly in the posterior corneal part causing distortion of the posterior corneal surface. LCD variant/H626 mutation.

All corneal changes were hyperreflective in the time domain and spectral OCT scans, but the level of increased reflectivity was different for different types of corneal dystrophies and increased in the following order: lattice corneal dystrophy, Thiel-Behnke corneal dystrophy, granular corneal dystrophy type I and granular corneal dystrophy type II. The opacities also were different in shape and pattern: from diffuse areas of increased reflectivity in lattice corneal dystrophy type I to focal, distinct and hyperreflective opacities in granular corneal dystrophies type I and II (Figure 1B,C,E,F,H,I and Figure 2B,D,F).

When comparing images of time-domain and spectral domain OCT, we noticed that all changes revealed on spectral swept source OCT scans were also visible on time-domain OCT. That makes both techniques useful for establishing the phenotype features of each TGFBI corneal dystrophy and monitoring the progression of the disease. The advantage of spectral swept source OCT over time-domain OCT scans is more distinct visualization of the Bowman’s layer area and the deposits located under the epithelium (Figure 1B,C,E,F,H,I).

### Phenotypic heterogeneity

**Figure 3:**
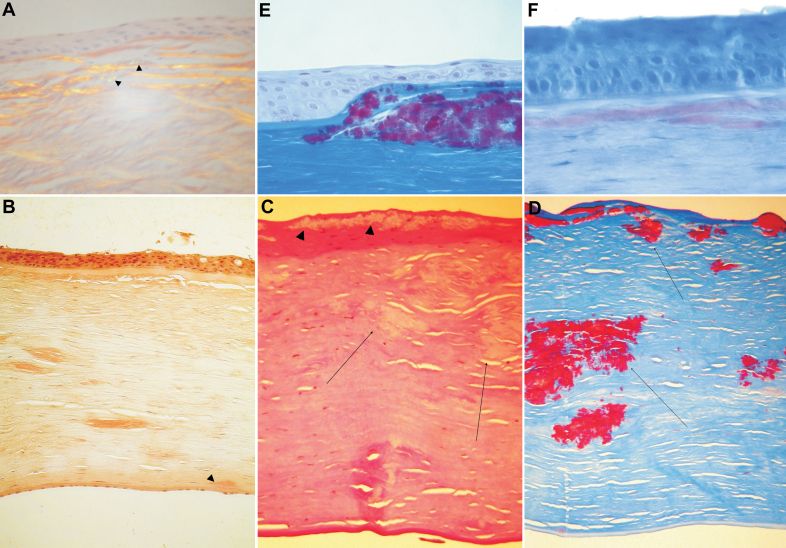
Representative images of histopathologic analysis of four corneal sections – three after penetrating keratoplasty and one after deep anterior lamellar keratoplasty. **A**: Section of the cornea after deep anterior lamellar keratoplasty. Male patient (F1; 37 years old). Green birefringence is visible with a polarizing filter (arrowheads). Stromal deposition of amyloid substance in anterior corneal part distorts the architecture of the corneal lamellae. The absence of Bowman’s layer and thinning of the epithelium are noticeable. LCDI/R124C mutation. **B**: Section of the cornea after penetrating keratoplasty. Congo red stain. Female patient (F2; 45 years). Deposits throughout the corneal stroma stain positive with Congo red. Note the deep, posterior corneal location of the deposit (arrowhead). LCD variant/H626 mutation. **C**: Section of the cornea after penetrating keratoplasty. PAS stain. Female patient (F4; 53 years). Note the absence of Bowman’s layer and the distorted epithelium in correspondence of the granular deposits (arrowheads). There are several granular deposits throughout the corneal stroma (arrows). GCDI/R555W mutation. **D**: Section of the cornea after penetrating keratoplasty. PAS stain. Female patient (F4; 53 years). Masson trichrome stain. Section of the cornea showing the absence of Bowman’s layer and the absence of the epithelium in correspondence with the Masson trichrome – positive granular deposits (arrows). GCDI/R555W mutation. **E**: Section of the cornea after penetrating keratoplasty. Masson trichrome stain. Female patient (F9; 44 years). Note that the granular deposits are placed under the thinner epithelium, thus taking the place of the former Bowman’s layer. GCDII/R124H mutation. **F**: Section of the cornea after penetrating keratoplasty. Congo red stain. Female patient (F9; 44 years). Note the Congo red positive deposits in the anterior corneal stroma. GCDII/R124H mutation.

Patients with TGFBI mutations R124C and R555W were characterized by a homogenous phenotype. The corneal phenotype of patients with the R555W mutation (families F4, F10, and F11) was characterized by granular crumb-like deposits in the central cornea, with age becoming located deeper in the stroma. In OCT scans, focal distinct opacities of increased reflectivity corresponding to corneal deposits were visible (Figure 1A-C). Two available corneal buttons revealed the interruption or local absence of Bowman’s layer, irregularity of the epithelium in correspondence with the superficial granular deposits and multiple granular deposits in the corneal stroma (Figure 3C,D). The corneal phenotype of patients with the R124C mutation (Families F1, F3, F5, F6, F12, F13, F14, and F15) was characterized by fragile, multiple lattice lines in the anterior to mid stroma in the central cornea with diffuse areas of increased reflectivity on corresponding OCT scans (Figure 2A,B). Five available corneal buttons revealed deposits that stained positive with Congo red. Green birefringence was visible with a polarizing filter (Figure 3A). Accompanying changes involved thinning of the epithelium and interruption of Bowman’s layer.

In the case of patients with R124H and R555Q mutations, we could not assess interfamilial heterogeneity, because there was only 1 family of each type. The phenotype of the R555Q mutation (family F8) was characterized by a “honeycomb” pattern of corneal deposits in the anterior corneal part. The changes visible on the OCT scans were limited to the anterior corneal stroma and the area of Bowman’s layer (Figure 1G-I). Since no members of family F8 underwent keratoplasty, the corneal button for histologic analysis was not available. The corneal changes in family F9 with the R124H mutation involved granular-, star- and disc-shaped stromal opacities without lattice lines. The opacities were highly reflective on OCT scans (Figure 1D-F). Histologic analysis revealed multiple granular deposits stained positively with Masson trichrome and a few sub Bowman’s layer Congo red positive amyloid deposits (Figure 3E,F).

Patients with the H626R mutation (Families F2 and F7) revealed interfamilial heterogeneity (Figure 2C-F). Corneal changes were asymmetric in both families. On the slit-lamp exam, a few thin, fragile lattice lines were located centrally in patients from family F2, while the lines were thick, distinct, extended to limbus and accompanied by stromal haze in patients from the family F7. OCT scans revealed that corneal opacities were located deep in the stroma in both families, even with deformation of the posterior corneal surface in family F7 (Figure 2C-F). The examination of one corneal button of family F2 confirmed the deep location of Congo red positive deposits in the corneal stroma (Figure 3B).

## Discussion

The development of molecular analysis has changed our understanding of corneal dystrophies. In 1997, Munier et al. [27] reported that mutation of one gene (*TGFBI*) caused dystrophies of different corneal layers. Consequently, this finding rendered classification based on corneal phenotype archaic, and it was replaced by the new IC3D system in 2008 [1].

Mutational and phenotypic variability has previously been reported, especially in different atypical forms of lattice dystrophy. Recently, the phenotypic heterogeneity was also described for R124C and R555W mutations [14-20,28,29]. The most recent studies suggested greater genetic heterogeneity than is currently known in this group of disorders [20,21,28,29]. This raises the question whether classification of the corneal stromal dystrophies should be based primarily on phenotype or on genotype. To answer that question, phenotype-genotype correlation reports from different ethnic groups, which have been studied widely in recent years, are needed [2-5,14-20,28-31].

In this first comprehensive report of TGFBI mutations covering a population in Poland, we found the lattice phenotype was dominant compared to the granular one (41:16 patients; lattice:granular). We identified five distinct mutations responsible for TGFBI corneal dystrophies (R124R, R124H, R555W, R555Q, and H626R). All those mutations were previously described. There was a strong genotype-phenotype correlation in the case of R124R (families F1, F3, F5, F6, F12, F13, F14, and F15) and R555W (families F4, F10, and F11) mutations, while there was a distinct phenotypic heterogeneity in the case of the H626R mutation (families F2 and F7). The mutation was first described by Stewart et al. [32] and Clair-Florent et al. [33] in 1999. Previous authors reported delayed disease onset and asymmetry of corneal changes [32-36]. The lattice lines were described as thicker and more distinct than in LCD type 1, which resembles the phenotype of our family F7. None of the previous authors revealed such deep deposits location that they caused the deformation of the posterior corneal surface as observed on OCT images of affected members from family F7 (Figure 2F). However, previous reports were based on slit-lamp and histopathologic findings, while in our study the deep corneal deposits affecting the posterior corneal surface were revealed exclusively based on OCT exams, not visible on the slit-lamp exam.

In our study, the phenotype of Avellino corneal dystrophy (family F9) differed from the classical form containing both granular and lattice deposits on the slit-lamp exam. In the case of family F9, no lattice lines were detectable on the slit-lamp and OCT exams (Figure 1D-F). Such heterogeneity was previously reported [37]. In our case, the diagnosis of GCDII was defined by revealing subepithelial Congo red positive deposits (Figure 3E,F) and confirming the presence of the R124H mutation. This underlines the need for precise phenotype analysis and *TGFBI* screening for definite diagnosis and classification of corneal dystrophies.

The current IC3D classification system contains the description of each corneal dystrophy based on slit-lamp, confocal microscopy, histopathology and transmission electron microscopy combined with the results of genetic analysis. In recent years, reports underlying the role of anterior segment optical coherence tomography in visualization of corneal morphology in corneal dystrophies have been published [38-40]. However, most papers were based on case reports of a single corneal dystrophy type, and the exam was performed by one OCT system. We confirmed the utility of time-domain and spectral OCT systems in establishing the characteristic features and the depth of corneal changes. Analyzing the depth of corneal deposits is one of the most important factors in choosing a treatment method. Performing an OCT exam is surely more detailed than the slit-lamp exam in assessing Descemet membrane involvement. OCT examination was already proven to be useful in optical coherence tomography-guided phototherapeutic keratectomy or in selection of lamellar versus penetrating keratoplasty as the treatment procedure of choice [41,42]. The main advantages of spectral versus time-domain OCT are the ability to visualize early corneal changes affecting the Bowman’s layer area (Figure 1) and the possibility of creating three-dimensional images. Compared to the slit-lamp examination, OCT provides detailed information about the disease progression based on documented analysis of corneal opacities’ depth and extent. Compared to confocal microscopy, OCT acquires less detailed information with axial resolution to 5 µm, but provides information about the range of corneal opacities and measures corneal thickness. In contrast to histopathologic examination, OCT can be performed in the early stages of the disease, without the need for keratoplasty. The recent development of OCT shows that this technique is in continuous change. New OCT prototypes gain axial resolution of 2–3 µm, which is more than twice that of OCT devices already used [43]. By using these new OCT prototypes, we could improve non-contact corneal imaging in the future.

In conclusion, this study underlines the role of comprehensive phenotype-genotype analysis in TGFBI corneal dystrophies, describes the TGFBI mutation spectrum in a Polish population and reveals the phenotypic heterogeneity in the case of the H626R mutation.

This study also highlights the need for a combined corneal morphology and genetic assessment to establish the diagnosis of TGFBI corneal dystrophies.
